# Special Issue: Corrosion Properties and Mechanism of Steels

**DOI:** 10.3390/ma15196796

**Published:** 2022-09-30

**Authors:** Vít Křivý

**Affiliations:** Department of Building Structures, Faculty of Civil Engineering, VSB–Technical University of Ostrava, L. Podeste 1875, 708 00 Ostrava, Czech Republic; vit.krivy@vsb.cz; Tel.: +420-774-442-936

The economic losses caused by corrosion are estimated to be 3–5% of gross domestic product in developed countries. Corrosion losses include the costs of replacing damaged devices, products or constructions, backup solutions, corrosion allowance, corrosion protection systems, loss of productivity, environmental and health damages, etc. Corrosion damage affects products made of various metallic materials, but the main group of products are structures made of steel. In terms of maintaining the required service life of structures or equipment, it is necessary to understand the corrosion damage mechanism, evaluate the impact on reliable services and propose appropriate measures.

The issue of corrosion damage is considered by engineers both in the design phase of a steel structure and during its service life. When designing steel structures, it is in many cases necessary to accurately predict the corrosion damage of the designed structural components. For structures in service, the influence of corrosion damage on the load-bearing capacity and serviceability of the structural component is usually evaluated and then the residual service life is predicted or reconstruction measures are implemented. In all of the above cases, it is important to understand the corrosion mechanisms specific to the material and environmental conditions. Cooperation between corrosion specialists and engineers responsible for the design and operation of a technological equipment or building construction is also very important.

The Special Issue “Corrosion Properties and Mechanism of Steels” has been proposed as a means to present recent developments in the field, and for this reason the fourteen articles included touch different aspects of steel corrosion: a brief summary of the articles’ content is given in the following. A significant phenomenon in the focus area of this Special Issue is the research on corrosion properties and mechanisms of steel pipelines. Half of the articles published in the Special Issue are devoted to this area.

The issue of corrosion processes in buried pipelines is covered in two papers prepared by Chung et al. [1,2]. A number of specific corrosion tests were carried out to investigate the synthetic soil corrosion of a pre-buried pipeline in [1]. Following the experimental test results, an empirical equation for the optimized CP current requirement, according to the pipeline service time, was derived. This equation can be applied to any corroded pipeline. External damage to buried pipelines caused by corrosive components in soil solution is studied in [2]. Increased attention is paid to the influence of the combination of pH, chloride and sulfate by using a statistical method according to the design of the experiment. The output of the used statistical methods is an equation that calculates the corrosion current density as a function of pH, chloride and sulfate concentration. Stray current corrosion in buried pipelines is investigated by Kang et al. [3]. In the article, as a countermeasure against stray current corrosion, calcareous depositions were applied to reduce the total amount of current flowing into pipelines and to prevent corrosion. The study examined the reduction of stray current corrosion via the formation of calcareous deposit layers, composed of Ca, Mg and mixed Ca and Mg at the current inflow area. Long-term corrosion mechanisms for steel pipelines in a soil environment are studied by So et al. [4] using electrochemical acceleration methods. Galvanostatic testing allows for accelerating the surface corrosion reactions through controlling the impressed anodic current density. However, a large deviation from the equilibrium state can induce different corrosion mechanisms to those in actual service. Therefore, applying a suitable anodic current density is important for shortening the test times and maintaining the stable dissolution of steel. To calibrate the anodic current density, galvanostatic tests were performed at four different levels of anodic current density and time to accelerate a one-year corrosion reaction of pipeline steel.

The issue of steel pipe failures is covered in the following two articles [5,6]. Lee et al. [5] investigated the cause of failure of a low-carbon steel pipe meeting standard KS D 3562 (ASTM A135) in a district heating system. In the groundwater environment outside of the pipe, localized corrosion occurred due to crevice corrosion by aluminum inclusions, and localized corrosion was accelerated by the large fraction of pearlite around the aluminum inclusions, leading to pipe failure. Witek [6] investigated the burst pressure and structural integrity of a steel pipeline based on in-line inspection results. Special attention was paid to the evaluation of data provided from the diagnostics using an axial excitation magnetic flux leakage technology in respect to multiple defects grouping. A specific corrosion environment of oilfield tubing and casing was studied by Dou et al. [7]. The high pressure and high temperature flow solution containing various gases and Cl^−^ ions significantly affects the corrosion processes. The high temperature corrosion conclusions provide references for the anticorrosion construction work of downhole pipe strings.

Several articles published in this Special Issue present detailed studies of specific corrosion processes of steel components used in mechanical engineering or in civil engineering industry. The effect of sulfate ions on galvanized post-tensioned steel corrosion in alkaline solutions is studied by Bonilla et al. [8]. The behavior of galvanized steel exposed to strong alkaline solutions with a fixed concentration of sulfate ions of 0.04 M is studied in detail. The coatings formed on stainless steel X20Cr13 were investigated by Kucharczyk et al. [9]. The article reports the results of the examination of the protective properties of silane coatings based on vinyltrimethoxysilane and ethanol, doped with the following electrolytes: acetic acid, lithium perchlorate LiClO_4_, sulphuric acid H_2_SO_4_ and ammonia NH_3_. Park et al. [10] examined the hydrogen-induced cracking caused by galvanic corrosion of an ASTM A516-65 steel weld in a wet sour environment using a combination of a standard immersion corrosion test, electrochemical analyses and morphological observation of the corrosion damage. Wang et al. [11] investigated the effects of Ti and Cu addition on inclusion modification and corrosion behavior in the simulated coarse-grained heat-affected zone of low-alloy steels by using an in situ scanning vibration electrode technique, a scanning electron microscope/energy-dispersive X-ray spectroscopy and an electrochemical workstation. The corrosion feature of Q235B steel in desulfurization solution is studied by Gong et al. [12]. The research results presented in the article are related to the tightening of marine diesel engine emission standards. In the article by Li et al. [13], a new mechanical-based experimental method is proposed to determine the corrosion initiation and subsequent corrosion behavior of steel in simulated concrete pore solutions. The proposed experiment is used to investigate the corrosion of the steel wire under various different conditions and to examine the effects of pre-stress level in steel wire, passivation time of steel wire, composition and concentration of simulated concrete pore solution on the corrosion initiation and the subsequent corrosion development in the steel wire. Křivý et al. [14] evaluated the static and corrosion performance of bolted lap joints in long-term operating towers. The article deals with the load-bearing capacity and durability of power line lattice towers designed from weathering steel. The design measures that can be applied in the design of new lattice towers are introduced in the article as well.
